# Effects of High-Dose Vitamin D Supplementation and Physical Exercise on Vitamin D Metabolites in Professional Football Players: A Pilot Study

**DOI:** 10.3390/nu18010175

**Published:** 2026-01-05

**Authors:** Anna Książek, Aleksandra Zagrodna, Konrad Kowalski

**Affiliations:** 1 Department of Biological Principles of Physical Activity, Faculty of Physical Education and Sports, Wroclaw University of Health and Sport Sciences, 51-612 Wroclaw, Poland; 2 Department of Bioenergetics and Exercise Physiology, Medical University of Gdańsk, 80-211 Gdańsk, Poland; 3 Masdiag Laboratory, 01-882 Warsaw, Poland

**Keywords:** 24,25-(OH)_2_D_3_, 3-*epi*-25-(OH)D_3_, dietary supplement, physical performance, athletes

## Abstract

**Background/Objectives**: Vitamin D plays an important role in muscle metabolism and recovery, yet its kinetics during and after football-specific physical activity remain poorly understood. Therefore, this study aimed to determine whether physical effort during a football match influences the concentration of vitamin D metabolites and to explore the effect of a single high-dose cholecalciferol supplementation combined with physical exercise on the levels of vitamin D metabolites in professional football players. **Methods**: Twenty professional football players participated in a three-phase, randomized placebo-controlled pilot study. Baseline fitness and blood samples were collected, followed by pre- and post-match measurements during two games. In the final phase, half of the players received a single 500,000 IU dose of vitamin D_3_ before a simulated match. Blood samples were collected before and after each session to analyze vitamin D metabolites using the isotope-dilution liquid chromatography–tandem mass spectrometry (ID-LC-MS/MS) method. **Results**: Physical exercise during the football match significantly increased serum concentrations of 25-(OH)D_3_, 24,25-(OH)_2_D_3_, and 3-*epi*-25-(OH)D_3_ (by up to 25%, *p* < 0.001). Following supplementation, these effects were further amplified, with 25-(OH)D_3_ rising by 98% and 3-*epi*-25-(OH)D_3_ by 424% (*p* < 0.001). Significant alterations in vitamin D metabolite ratios after exercise and supplementation suggest enhanced metabolic turnover and dynamic regulation of vitamin D pathways in response to physical effort. **Conclusions**: Football-specific physical activity appears to stimulate the release of vitamin D metabolites. High-dose cholecalciferol supplementation was well tolerated and may rapidly increase vitamin D status in professional athletes. These findings may have implications for optimizing recovery and performance, though larger trials are needed.

## 1. Introduction

The active form of vitamin D regulates key physiological processes by interacting with the vitamin D receptor (VDR) [1], and is essential for muscle function, bone health, and recovery [2]. A review conducted by Harju et al. [3] showed that over 30% of athletes had 25-(OH)D concentrations < 20 ng/mL, defined as vitamin D deficiency [4]. Growing evidence supports a role of vitamin D supplementation in mitigating exercise-related inflammation, which could enhance recovery efficiency and minimize the long-term injury risk among athletes [5]. However, there is still no consensus on the issue of standardized norms for vitamin D concentrations and the optimal dose of supplementation in athletes remains a subject of ongoing debate.

The primary storage site for vitamin D is adipose tissue, and physical exercise stimulates the release of vitamin D from adipocytes, contributing to an increase in its blood levels [6]. Mawer et al. [7] documented that skeletal muscles are also capable of both storing and releasing 25-(OH)D. The presence of enzymatic activity of 25-hydroxyvitamin D-1α-hydroxylase (CYP27B1) and cytochrome P450 family 24 subfamily A member 1 (CYP24A1) in skeletal muscle tissue suggests the potential for localized vitamin D metabolism and autocrine regulation within muscle cells [8,9]. It has been demonstrated that vitamin D release from muscle tissue can be stimulated by physical exercise, with the intensity of the activity influencing the magnitude of this effect [10].

Thus far, the majority of studies have focused on the impact of a single bout of physical exercise on 25-(OH)D concentration [10,11]. However, there is a limited number of studies [12] that have investigated other vitamin D metabolites, such as 24,25-(OH)_2_D and 3-*epi*-25-(OH)D_3_. These metabolites are generally considered biologically inactive; nonetheless, certain studies have demonstrated their significant association with skeletal muscle function in relation to athletic performance [13], and prolonged, uninterrupted physical effort [12]. To date, no studies have investigated how acute, football-specific exercise interacts with single high-dose vitamin D supplementation to modulate vitamin D metabolites.

In light of the above, the primary aim of the study was to evaluate the acute effects of a football league match on serum concentrations of vitamin D metabolites in professional players. The secondary objective was to examine how these metabolites respond to high-dose vitamin D_3_ supplementation combined with football-specific exercise during an intra-squad game. The tertiary aim was to evaluate the physiological response of professional football players to a single high-dose vitamin D_3_ supplementation.

## 2. Materials and Methods

### 2.1. Subjects

The study included healthy, male professional football players aged 18–35 years, who were active members of a first-division Polish football team. The characteristics of athletes are presented in Table 1. The team competed and trained regularly at latitudes between 50° and 54° N. Eligible participants had no recent injuries, were fully engaged in regular training and match activities, and had not used vitamin D or calcium supplements for at least three months prior to the start of the study. Athletes were excluded if they reported any current musculoskeletal or systemic injury, or any chronic illness or pharmacological treatment that could interfere with vitamin D metabolism. Additional exclusion criteria included refusal to provide written informed consent and non-compliance with study procedures.

### 2.2. Experimental Overview

The research experiment comprised three stages. In the first stage (P1), a general blood test was performed to assess the athletes’ health status, skinfold thickness was measured, and aerobic fitness was evaluated using the 30–15 Intermittent Fitness Test, which was used to estimate maximal oxygen uptake (VO_2_max). Details regarding the procedures for general blood test, measuring skinfold thickness and assessing aerobic fitness have been described in our previous articles [13,14,15]. In the second stage (P2), the athletes participated in a league match (LM). Ten players were actively involved in the game (P) and ten players did not participate (NP) (goalkeepers were excluded from the experiment). Blood samples were collected two hours before (T1) and immediately after the match (T2). In the third stage of the experiment (P3), participants were randomly allocated to the supplementation group (SGP) or the placebo group (PGP). Two days after supplementation, an intra-squad game (ISG) was played, and blood samples were collected before supplementation (T3), two hours before ISG (T4), and immediately after ISG (T5). This study was registered at ClinicalTrials.gov (Identifier: NCT07310329) on 30 December 2025.

### 2.3. Vitamin D Supplementation

Athletes were randomly allocated in a 1:1 ratio to the supplementation group (SGP) or the placebo group (PGP) using a computer-generated randomization procedure. The randomization was single-blind, as athletes were unaware of whether they received vitamin D or placebo. To maintain balance between playing positions, players from different field formations (excluding the goalkeeper) were distributed across both groups. Each participant in SGP received a single high dose of vitamin D_3_ (500,000 IU; Vigantol Oil, P&G Health, Schwalbach am Taunus, Germany) 48 h before ISG. The placebo group received pure vegetable oil designed to match the vitamin D_3_ solution in appearance and viscosity. Blinding procedures ensured that participants were unaware of group allocation or formulation differences.

### 2.4. Vitamin D and Calcium Dietary Intake

Assessment of vitamin D and calcium intake among the study participants was conducted using a 7-day food diary. Prior to the start of the first phase of the experiment, participants received detailed instructions on how to accurately record their daily intake of food and beverages, including both main meals and snacks. The diary entries were required to include the name of the food product and the precise quantity consumed, expressed in grams, milliliters, or household measures (e.g., tablespoons, cups). The intake of vitamin D and calcium was analyzed using the Dieta 6.0 software (National Institute of Public Health—NIH, Warsaw, Poland).

### 2.5. Measurements of Vitamin D Metabolite Levels

For the determination of vitamin D metabolites, an isotopic dilution method is used together with high-performance liquid chromatography coupled to tandem mass spectrometry (ID-LC-MS/MS) technique. The main circulating metabolites of vitamin D are analysed including: 25(OH)D_3_, 25(OH)D_2_, 3-*epi*-25(OH)D_3_ and 24,25(OH)_2_D_3_. A combination of protein precipitation and derivatisation is used to prepare the serum sample (50 µL). Double protein precipitation with acetonitrile is used where isotopically labelled internal standards (13C_5_-25(OH)D_3_, 2H_6_-24,25(OH)_2_D_3_, 2H_3_-25(OH)D_2_ and 2H_3_-3-*epi*-25(OH)D_3_) are added in the first step. Each time the precipitation was carried out for 10 min at room temperature at shaking speeds of 1100 and 3000 rpm, respectively. After centrifugation of the precipitate, the transferred supernatant is evaporated in a stream of nitrogen (12 L/min, 15 min at 55 °C). Derivatization using the Diels-Adler reaction is carried out by adding the reagent DAPTAD (4-(4′-dimethylaminophenyl)-1,2,4-triazoline-3,5-dione) in ethyl acetate to the dry residue and the reaction is carried out for 30 min (RT, 450 rpm). After re-evaporation, the dry residue is dissolved in a methanol: water mixture (1:1) and analysed using LC-MS/MS. Chromatographic separation of vitamin D metabolites is carried out using a thermostated column (40 °C) in reverse phase mode (Agilent, Zorbax Eclipse XDB-C18, 80 Å, 4.6 × 50 mm, 1.8 µm; Santa Clara, CA, USA) at a flow rate of 0.8 mL/min in a linear gradient (from 50%:50% to 2%:98%) of water and acetonitrile both with 0.1% formic acid as mobile phases. Analysis is performed on a Shimadzu NexeraXR LC-20AD XR liquid chromatograph (Shimadzu, Kyoto, Japan) using a CTC PALxt autosampler (CTC Analytics, Zwingen, Switzerland) and coupled to a Sciex QTRAP5500 tandem mass spectrometer equipped with an electrospray ion source (TurboV Ion Source, Sciex, Framingham, MA, USA). Ions are observed in positive ion mode at 650 °C with electrospray generated at 4500 V and nebuliser (GS1) and desiccant (GS2) gas flows of 45 psi and 50 psi respectively. Unique fragmentation reactions are observed in single reaction monitoring (SRM, Q1 mass/Q3 mass) mode for both analytes (25(OH)D_3_ and *epi*-25(OH)D_3_, 619.5/341.2; 25(OH)D_2_, 631.5/341.2, 24,25(OH)_2_D_3_ 635.5/341.2) and internal standards (13C_5_-25(OH)D_3_, 624.5/341.2; 2H_3_*-epi-*25(OH)D_3_, 622.5/344.2; 2H_3_-25(OH)D_2_, 634.5/344.2; 2H_6_-24,25(OH)_2_D_3_ 641.5/341.2) with ion transmission parameters including declastering potential (DP) and collision energy (CE) of 120 eV and 36 eV, respectively. Quantitative analysis by isotopic dilution is performed using a 7-point calibration curve prepared by enriching vitamin D free serum (VD-DC Mass Spect Gold^®^, Gold West Biologicals, Temecula, CA, USA) with certified reference standards of the vitamin D metabolites. The method presented has been fully validated and is subject to inter-laboratory control within the DEQAS (Vitamin D External Quality Assessment Scheme) programme. The method has satisfactory sensitivity at a limit of quantification (LOQ) of 0.1 ng/mL and appropriate analytical parameters (varying between analytes) including linearity (R2 greater than 0.995) recovery (95–98%), precision (less than 5%) and accuracy (relative bias less than 10%).

### 2.6. Characterization of Effort Performed During the LM and the ISG

In the second phase of the experiment (P2), the study was conducted during a league football match in April. The team played in a 1-4-4-2 formation, consisting of four defenders, four midfielders, and two forwards positioned on the same line. The match took place at a football stadium with a natural grass surface. The game commenced at 8:30 p.m., with an air temperature of 18 °C and wind speeds ranging from 3 to 6 m/s. Data collection was performed using the Catapult system, which included 14 MinimaxX S4 receivers and Sprint software (Catapult Sports version 5.1, Melbourne, VIC, Australia). The receivers were checked and charged, and approximately 15 min before the match, the devices were activated to allow for the determination of geographical positions. Prior to the match, the players completed a 10-min warm-up followed by dynamic ball drills, including rondo passing, triangle passing with movement, slalom dribbling through cones, one-touch passing on the move, and short sprints with the ball followed by goal attempts. During the final stage of the pre-match warm-up, players were fitted with appropriately sized vests in which the Catapult receivers were inserted. The exact start and end times of the first and second halves of the training match were recorded. The players covered a total distance of 114.5 km over two halves. The majority of the movement involved walking (37.8 km) and jogging (26.8 km). The average running speed of the outfield players during the match was 119.3 m/min. The midfielders were the most active formation, with wide midfielders covering 115.75 m/min and central midfielders 113.0 m/min. The procedure described above was repeated during the third phase of the experiment (P3). This phase was conducted during ISG in June. The team employed the same 1-4-4-2 formation. The match took place at a football stadium with a natural grass surface. As before, the players performed a 10-min warm-up followed by dynamic ball exercises. The ISG began at 5:00 p.m., with an air temperature of 28 °C and wind speeds ranging from 4 to 8 m/s. The players covered approximately 104 km, with a 3.5 km reduction in activity during the second half. The majority of the distance was covered through walking (38.5 km) and jogging (25.9 km), accounting for 64% of the total distance travelled during the ISG. The midfielders were the most active formation, with wide midfielders covering 112.4 m/min and central midfielders 107.69 m/min. The least active players were central defenders, who averaged 92.3 m/min.

### 2.7. Statistical Analysis

All data are expressed as mean ± standard deviation (SD). The normality of distribution for each variable was assessed using the Shapiro–Wilk test. Between-group differences in baseline characteristics were evaluated using Student’s *t*-test for normally distributed variables and the Mann–Whitney U test for non-parametric data. Within-group changes before and after the league match (P2) were analyzed using the Wilcoxon signed-rank test, whereas between-group comparisons at individual time points were examined with the Kruskal–Wallis test. During the supplementation phase (P3), a two-way repeated-measures ANOVA (2 groups × 3 time points) was applied to evaluate the effects of group (supplementation [SGP] vs. Placebo [PGP]) and time (T3, T4, T5) on serum vitamin D metabolite levels. When a significant interaction was observed, Tukey’s post hoc procedure was performed to identify specific differences between subgroups. Effect sizes were estimated using eta squared (η^2^) and interpreted as small (≥0.01), moderate (≥0.06), or large (≥0.14). All statistical analyses were conducted in Statistica version 13 (TIBCO Software Inc., Palo Alto, CA, USA). A *p*-value ≤ 0.05 was considered statistically significant.

## 3. Results

Results from 20 participants were included in the data analysis. No significant differences were observed between the supplementation (SGP) and placebo (PGP) groups in baseline characteristics, including age, body composition, serum 25-(OH)D levels, and biochemical markers such as hemoglobin, glucose, testosterone, cortisol, ferritin, adjusted calcium, and creatine kinase. Additionally, no differences were found in aerobic capacity (VO_2_max) or dietary intake of vitamin D and calcium (Table 1). The baseline characteristics of all group are shown in Table 1. The mean serum 25-(OH)D level was 22.1 ± 3.2 ng/mL. Assuming serum 25-(OH)D levels in the range of 30–50 ng/mL to be the physiological norm [4], we found that 90.0% of the participants exhibited values below the optimal range for vitamin D status.

### 3.1. Changes in Vitamin D Metabolite Concentrations Induced by Physical Activity During LM

Table 2 presents the changes in vitamin D metabolite concentrations in athletes who participated in LM and those who did not. The study results indicated that the concentrations of 25-(OH)D_3_, 24,25-(OH)_2_D_3_ and 3-*epi*-25-(OH)D_3_ significantly increased in athletes who played in a league match, by 13% (*p* < 0.001), 25% (*p*< 0.001), 16% (*p* < 0.05) respectively. Similar changes were observed in athletes who did not participate in the league match, with 25-(OH)D_3_ levels increasing by 9% (*p* < 0.001) and 3-*epi*-25-(OH)D_3_ by 5% (*p* < 0.05). Data analysis revealed a 11% decrease (*p* < 0.05) in the 25-(OH)D_3_:24,25-(OH)_2_D_3_ ratio and 11% increase (*p* < 0.05) in the 24,25-(OH)_2_D_3_:25-(OH)D_3_ in athletes who played in the league match. The findings demonstrated that the vitamin D metabolite ratio significantly differed between athletes who participated in the match and those who did not. These differences were observed for 25-(OH)D_3_:3-*epi*-25-(OH)D_3_ in T1 (*p* < 0.05) and T2 (*p* < 0.05) (Table 2).

### 3.2. Changes in Vitamin D Metabolite Levels in the SGP and PGP Groups Induced by Physical Exercise During ISG

**Figure 1:**
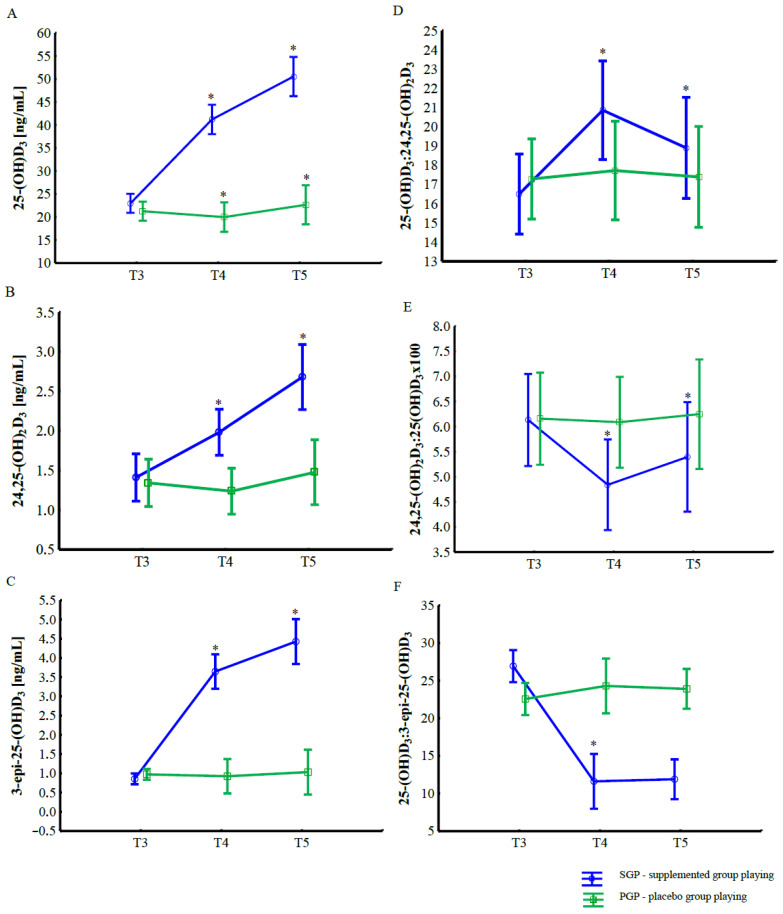
Changes in serum vitamin D metabolite concentrations and metabolite ratios in the supplemented group playing (SGP) and placebo group playing (PGP) induced by high-dose vitamin D_3_ supplementation and football-specific physical exercise during the intra-squad game (ISG); (**A**) 25-(OH)D_3_, (**B**) 24,25-(OH)_2_D_3_, (**C**) 3-*epi*-25-(OH)D_3_, (**D**) 25-(OH)D_3_:24,25-(OH)_2_D_3_ ratio, (**E**) 24,25-(OH)_2_D_3_:25-(OH)D_3_ ×100 ratio, and (**F**) 25-(OH)D_3_:3-*epi*-25-(OH)D_3_ ratio; Data are presented as mean ± SD. * indicates statistically significant differences between time points (T3, T4, and T5) within groups at *p* < 0.001; SGP—supplemented group playing; PGP—placebo group playing.

Changes in serum concentrations of vitamin D metabolites during the supplementation and intra-squad game (ISG) phase are presented in Figure 1 and Table 3. The two-way ANOVA (2 groups × 3 time points) revealed a significant effect of group, time, and their interaction for all analyzed metabolites: 25-(OH)D_3_, 24,25-(OH)_2_D_3_, and 3-*epi*-25-(OH)D_3_ (*p* < 0.001 for all comparisons; Table 3).

Regardless of supplementation, a significant increase was observed in serum 25-(OH)D_3_, 24,25-(OH)_2_D_3_, and 3-*epi*-25-(OH)D_3_ concentrations after the ISG compared with pre-game values. However, these changes were substantially greater in the supplemented group (SGP) than in the placebo group (PGP). In the SGP, serum 25-(OH)D_3_ increased by approximately 98% following supplementation (T3 vs. T4, *p* < 0.001) and remained elevated after exercise (T5, *p* < 0.001). Similarly, concentrations of 24,25-(OH)_2_D_3_ and 3-*epi*-25-(OH)D_3_ increased by 41% and 424%, respectively, from baseline to post-supplementation, with further elevations observed after the intra-squad match (*p* < 0.001 for all comparisons).

In contrast, no significant changes were found in the placebo group across time points. Between-group comparisons demonstrated that 25-(OH)D_3_, 24,25-(OH)_2_D_3_, and 3-*epi*-25-(OH)D_3_ levels were significantly higher in SGP group at all measurement points (T3–T5, *p* < 0.001).

No significant main or interaction effects were observed for the ratios 25-(OH)D_3_:24,25-(OH)_2_D_3_ and 24,25-(OH)_2_D_3_:25-(OH)D_3_. However, a significant group × time interaction was detected for the ratio 25-(OH)D_3_:3-*epi*-25-(OH)D_3_ (p < 0.001), which decreased markedly after supplementation and exercise in the SGP compared with the PGP.

## 4. Discussion

The objective of this study was to evaluate the impact of physical exercise performed during football matches and high-dose vitamin D_3_ supplementation on the concentrations of vitamin D metabolites in professional football players. Unlike most previous research, which has focused almost exclusively on total 25-(OH)D, our study also examined other metabolites such as 24,25-(OH)_2_D_3_ and 3-*epi*-25-(OH)D_3_, whose roles in muscle physiology and athletic performance remain poorly understood. To the best of our knowledge, this is one of the first studies to investigate the combined effect of football-specific physical effort and high-dose vitamin D_3_ supplementation on the levels of various vitamin D metabolites.

Our findings indicate that physical effort during league match resulted in a significant increase in the concentrations of 25-(OH)D_3_ and 24,25-(OH)_2_D_3_ in athletes actively participating in matches. These outcomes are consistent with previous evidence indicating that physical activity can stimulate the release of vitamin D from adipose tissue [6] and skeletal muscle [10]. For instance, Sun et al. [11] demonstrated a significant increase in serum 25-(OH)D levels following 30 min of exercise (cycling at 70% VO_2_max) in young, healthy men and women, with the increase persisting up to 24 h post-exercise. This increase persisted both immediately post-exercise and up to 24 h afterward. Moreover, men exhibited higher 25-(OH)D levels both before and after exercise compared to women, which may be attributed to differences in body composition. Similarly, Dzik et al. [10] examined the impact of a single exercise session in adolescent boys and found that both VO_2_max aerobic tests on a cycling ergometer and the Wingate anaerobic test (WAnT) elevated 25-(OH)D_3_ levels, with the most pronounced effect observed following high-intensity interval training (WAnT). Vitamin D plays a pivotal role in skeletal muscles, as confirmed by studies on both animal models and humans [16]. Mawer et al. [7] observed that vitamin D is predominantly stored in adipose tissue and muscle, from where it is gradually released, potentially influencing its concentration in the body and its role in muscle metabolism. While vitamin D stored in fat tissue is not easily accessible and becomes available primarily during fatty acid mobilization [17], skeletal muscles appear to have a more dynamic interaction with vitamin D. The changes observed in this study appear to show that physical exercise may modulate vitamin D metabolism, possibly due to increased utilization or synthesis in skeletal muscles or other tissues during physical activity. Our study also indicated changes in additional metabolites, including 24,25-(OH)_2_D_3_ and 3-*epi*-25(OH)D_3_, which have rarely been investigated in athletic populations. Notably, significant alterations were recorded in the ratios of vitamin D metabolites, including a 11% decrease in the 25-(OH)D_3_:24,25-(OH)_2_D_3_ ratio and an 11% increase in the 24,25-(OH)_2_D_3_:25-(OH)D_3_ ratio following LM activity in athletes actively participating in the game. These changes may reflect both the professional training status of our participants and the football-specific nature of the exercise protocol, although further research is needed to confirm this.

The link between vitamin D and the specific physical demands of football should also be highlighted. Football players perform activities that combine high-intensity sprints with prolonged moderate-intensity efforts [18,19], which may significantly influence vitamin D metabolism. High-intensity sprints predominantly recruit fast-twitch muscle fibers, which depend on optimal vitamin D levels [20]. On the other hand, prolonged moderate- or low-intensity activities (e.g., jogging, walking) performed during matches may depend on vitamin D’s role in calcium homeostasis and muscle contractions, crucial for sustaining endurance [21]. Studies have shown a prevalence of vitamin D deficiency among football players, as evidenced by multiple scientific publications [3,22,23]. Given these unique physical demands and the documented prevalence of vitamin D deficiency in football players training at northern latitudes, ensuring adequate vitamin D status may be particularly important for sustaining both high-intensity performance and recovery in this athletic population.

One of the noteworthy findings of this study was a substantial increase in 3-*epi*-25(OH)D_3_ concentrations (+424%) following high-dose vitamin D_3_ supplementation (500,000 IU), accompanied by a moderate rise in 25-(OH)D_3_ levels (+98%). This pronounced change may reflect enhanced epimerase activity, leading to the conversion of 25-(OH)D_3_ into its C-3 epimer, 3-*epi*-25-(OH)D_3_, which represents an alternative metabolic pathway of vitamin D [1]. Similar observations have been previously reported following vitamin D supplementation, for example by Mieszkowski et al. [12], who found that serum concentrations of 25-(OH)D_3_, 24,25-(OH)_2_D_3_, and 3-*epi*-25-(OH)D_3_ increased after an ultramarathon, regardless of supplementation status. This mechanism may therefore constitute part of a broader adaptive metabolic response to a sudden rise in substrate availability. Consistent findings were reported by Leszczyńska et al. [24], who observed a marked increase in 3-*epi*-25-(OH)D_3_ concentrations as early as the third day after a single oral dose of 120,000 IU of vitamin D_3_, suggesting that epimerization may function as an early catabolic mechanism protecting against vitamin D toxicity.

The biological significance of 3-*epi*-25-(OH)D_3_ remains incompletely understood; however, current evidence indicates that this metabolite exhibits lower affinity for both vitamin D-binding protein (DBP) and the vitamin D receptor (VDR), which results in a faster plasma clearance rate and reduced transcriptional activity [1]. Although some authors have not confirmed a relationship between 3-*epi*-25-(OH)D_3_ and indices of body composition or muscle strength [25], our previous findings [13] demonstrated a significant association between 3-*epi*-25-(OH)D_3_ levels and indices of power, strength, and vertical jump performance in indoor athletes. These data may suggest that 3-*epi*-25-(OH)D_3_ plays a specific role in regulating skeletal muscle function, particularly under conditions of increased metabolic demand and physical exertion.

Taken together, these findings support the hypothesis that 3-*epi*-25-(OH)D_3_ is not merely an inactive metabolic byproduct but may participate in the local regulation of muscle-related processes, especially during periods of high physical load. However, this remains a working hypothesis that requires further experimental verification. Future research involving athletes is warranted to determine whether the observed increase in 3-*epi*-25-(OH)D_3_ represents solely an adaptive and protective response or whether it also exerts a functional impact on muscle performance, recovery, and energy metabolism.

Our findings are in line with studies on Polish football players, where supplementation protocols using lower doses (5000–200,000 IU) [26,27] also effectively increased serum 25-(OH)D and in some cases improved anaerobic capacity [26]. To the best of our knowledge, this is the first study to examine a single high dose of vitamin D in athletes, as previous research has predominantly focused on lower doses ranging from 150,000 to 200,000 IU [12,28,29]. However, the response to this mega-dose was relatively limited, as evidenced by a less than 100% increase in 25-(OH)D_3_ levels (from 21.9 to 43.4 ng/mL). This limited effect may be attributed to the presumed role of skeletal muscle as a storage site for vitamin D, which likely restricts its direct impact on circulating metabolite levels. Notably, the high-dose vitamin D supplementation did not induce adverse effects such as hypercalcemia or gastrointestinal discomfort, supporting the safety of this supplementation strategy under controlled conditions. Various parameters were regularly monitored over a six-month period to ensure that participants did not develop any risk of hypercalcemia or hyperkalemia. The obtained results, combined with existing scientific knowledge, may indicate the need to revise current standards regarding vitamin D concentrations and supplementation doses for athletic populations. The specific nature of physical activity, particularly in a discipline like football, may require higher vitamin D levels, making general population recommendations insufficient. From a practical standpoint, these results emphasize the importance of individualized monitoring of vitamin D status in professional athletes. Regular assessment of 25-(OH)D and its metabolites may help optimize supplementation strategies, ensuring adequate levels to support muscle function and recovery without risk of excessive accumulation.

This pilot study presents several strengths. It is one of the first to explore the effects of a single high-dose vitamin D_3_ supplementation combined with football-specific physical effort on a broad range of vitamin D metabolites, including 24,25-(OH)_2_D_3_ and 3-*epi*-25-(OH)D_3_. The study was conducted on a homogeneous group of professional football players, using a randomized controlled design and highly sensitive analytical methods (ID-LC-MS/MS).

Despite these strengths, several limitations should be noted. The small sample size, short follow-up period, and inclusion of only male athletes from a single professional team limit the generalizability of our findings. Second, despite efforts to maintain standardized dietary intake and training loads, factors such as seasonality and dietary variability could still have influenced vitamin D status and its metabolites. Third, we did not assess functional outcomes such as markers of inflammation, recovery, or physical performance, which would help to contextualize the metabolic changes observed. Individual variability related to genetic or molecular factors was also not addressed. 

## 5. Conclusions

In conclusion, this pilot study suggests that skeletal muscles may act as a reservoir of 25-(OH)D_3_, with its release potentially stimulated by football-specific exercise. A single high dose of cholecalciferol appeared safe and was associated with increased concentrations of vitamin D metabolites in professional football players. For practitioners, these findings highlight the importance of monitoring vitamin D status throughout the season and raise the possibility that short-term corrective strategies, such as high-dose supplementation under medical supervision, could be considered during periods of intensive training or competition. However, given the small sample size, short follow-up, and exploratory design, these results should be interpreted with caution and regarded as hypothesis-generating. Larger, long-term studies are needed to confirm these observations and to determine whether current supplementation guidelines adequately reflect the unique physiological demands of football.

## Figures and Tables

**Table 1:** Participants’ characteristic.

	Mean ± SD		
	All (*n* = 20)	SGP (*n* = 10)	PGP (*n* = 10)
Age [years]	25.0 ± 4.0	26.0 ± 4.0	25.0 ± 4.0
Body weight [kg]	77.0 ± 6.5	77.1 ± 6.7	77.1 ± 5.2
Height [cm]	181.0 ± 6.0	181.0 ± 6.0	181.0 ± 5.0
Body fat [%]	6.6 ± 1.7	6.6 ± 1.5	6.8 ± 1.7
25-(OH)D_3_ [ng/mL]	22.1 ± 3.2	23.0 ± 3.7	21.3 ± 2.5
24,25-(OH)_2_D_3_ [ng/mL]	1.4 ± 0.4	1.4 ± 0.3	1.3 ± 0.6
3-*epi*-25-(OH)D_3_ [ng/mL]	0.9 ± 0.2	0.9 ± 0.1	1.0 ± 0.3
25-(OH)D_3_:24,25-(OH)_2_D_3_	16.9 ± 3.1	16.5 ± 1.8	17.3 ± 4.0
24,25-(OH)_2_D_3_:25-(OH)D_3_ × 100	6.1 ± 1.3	6.1 ± 0.7	6.2 ± 1.9
25-(OH)D_3_:3-*epi*-25-(OH)D_3_	24.8 ± 3.9	26.9 ± 3.3	22.6 ± 3.1
Hemoglobin [g/dL]	15.0 ± 0.6	15.0 ± 0.7	15.0 ± 0.6
RBC [mln/μL]	5.3 ± 0.3	5.3 ± 0.3	5.2 ± 0.2
WBC [tys/μL]	6.3 ± 1.1	6.3 ± 1.1	6.4 ± 1.2
Glucose [mg/dL]	89.6 ± 5.4	89.7 ± 5.7	90.3 ± 5.3
Testosterone [ng/dL]	619.3 ± 152.6	632.6 ± 161.7	663.3 ± 126.0
Cortisol [μg/dL]	17.6 ± 3.6	17.7 ± 3.6	16.6 ± 3.2
Ferritin [μg/L]	118.0 ± 103.2	115.9 ± 105.1	113.3 ± 114.2
ACa [mg/dL]	9.3 ± 0.6	9.3 ± 0.4	9.4 ± 0.3
CK [U/L]	312.0 ± 153.8	303. ± 154.9	301.9 ± 159.5
VO_2_max [mL/kg/min]	60.4 ± 4.6	59.8 ± 4.2	61.3 ± 4.1
Vitamin D intake [µg/d]	4.9 ± 2.9	4.7 ± 2.8	5.0 ± 2.9
Calcium [mg/d]	1162 ± 260	1121 ± 225	1205 ± 321

SGP—players who received vitamin D supplementation, PGP—players who received placebo; RBC—red blood cells, WBC—white blood cells, ACa—albumin-adjusted calcium, CK—creatine kinase, VO_2_max—maximal oxygen uptake.

**Table 2:** Changes in vitamin D metabolite concentrations and ratios induced by physical activity during LM.

	Mean ± SD			
	P (*n* = 10)		NP (*n* = 10)	
	T1	T2	T1	T2
25-(OH)D_3_ [ng/mL]	22.9 ± 4.3	25.8 ± 5.1 ***	23.4 ± 9.6	25.5 ± 9.6 **
24,25-(OH)_2_D_3_ [ng/mL]	1.6 ± 0.3	2.0 ± 0.4 ***	2.1 ± 1.3	2.0 ± 1.1
3-*epi*-25-(OH)D_3_ [ng/mL]	0.9 ± 0.1	1.0 ± 0.2 *	1.2 ± 0.7	1.3 ± 0.7 *
25-(OH)D_3_:24,25-(OH)_2_D_3_	14.7 ± 2.7	13.1 ± 1.7 *	12.4 ± 3.5	14.2 ± 4.8
24,25-(OH)_2_D_3_:25-(OH)D_3_ × 100	7.0 ± 1.2	7.8 ± 1.1 *	8.6 ± 2.0	7.6 ± 2.0
25-(OH)D_3_:3-*epi*-25-(OH)D_3_	25.7 ± 3.9	26.8 ± 5.4	20.9 ± 4.1 ^#^	21.3 ± 6.0 ^#^

LM—league match; P—playing group, NP—non-playing group; T1—Pre-match, T2—Post-match; Statistical significance: * *p* < 0.05; ** *p* < 0.01, *** *p* < 0.001; P—T1 vs. T2 and NP—T1 vs. T2; ^#^ *p* < 0.05; P T1 vs. NP T1 and between P T2 vs. NP T2.

**Table 3:** Two-way (2 groups × 3 repeated measures) ANOVA for serum concentrations of vitamin D metabolites and their ratios induced by football game and supplementation.

Variable	Effect	F	df	*p*	Effect Size (η^2^)
25-(OH)D_3_ [ng/mL]	GR	174.73	1	0.00	0.76
	TIME	42.90	2	0.00	0.61
	GR × TIME	37.52	2	0.00	0.58
24,25-(OH)_2_D_3_ [ng/mL]	GR	26.07	1	0.00	0.33
	TIME	9.84	2	0.00	0.27
	GR × TIME	6.27	2	0.004	0.19
3-*epi*-25-(OH)D_3_ [ng/mL]	GR	141.83	1	0.00	0.72
	TIME	42.24	2	0.00	0.61
	GR × TIME	41.03	2	0.00	0.60
25-(OH)D_3_:24,25-(OH)_2_D_3_	GR	1.85	1	0.18	0.03
	TIME	2.14	2	0.13	0.07
	GR × TIME	1.45	2	0.24	0.05
24,25-(OH)_2_D_3_:25-(OH)D_3_	GR	3.49	1	0.07	0.06
	TIME	1.08	2	0.35	0.04
	GR × TIME	0.90	2	0.41	0.03
25-(OH)D_3_:3-*epi*-25-(OH)D_3_	GR	36.68	1	0.00	0.40
	TIME	16.53	2	0.00	0.38
	GR × TIME	24.87	2	0.00	0.48

GR—group effect (supplemented vs. placebo); TIME—time effect (T3, T4, T5); GR × TIME—interaction effect between group and time; F—F-statistic; df—degrees of freedom; *p*—level of statistical significance; η^2^—eta-squared (effect size).

## Data Availability

The data presented in this study are available on request from the corresponding author due to privacy and ethical restrictions involving professional athletes.
